# Supplementary material for: Design strategy for serine hydroxymethyltransferase probes based on retro-aldol-type reaction
Source: Nat Commun. 2019 Feb 20;10:876. doi: 10.1038/s41467-019-08833-7 (PMC6382819; doi:10.1038/s41467-019-08833-7)
Supplement: Supplementary file 1 — Supplementary Information [file 41467_2019_8833_MOESM1_ESM.pdf]

Supplementary Information

Design Strategy for Serine Hydroxymethyltransferase Probes Based on Retro-aldol Type Reaction

Nonaka *et al.*

Supplementary figures

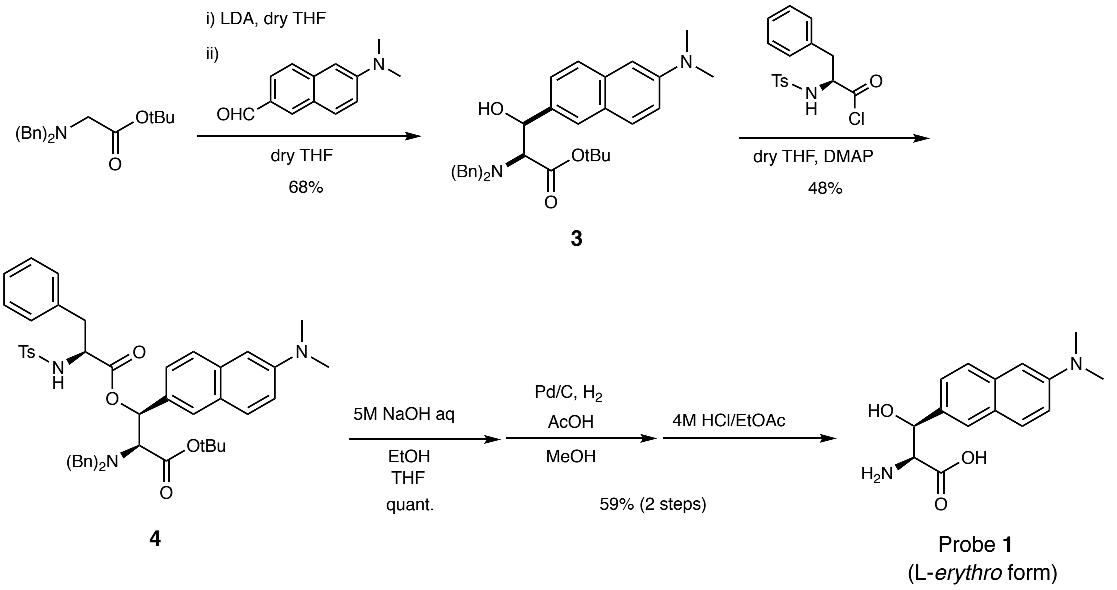

Supplementary Figure 1 | Synthesis of the SHMT fluorescent probe 1. The detailed procedures are described in Supplementary Methods section.

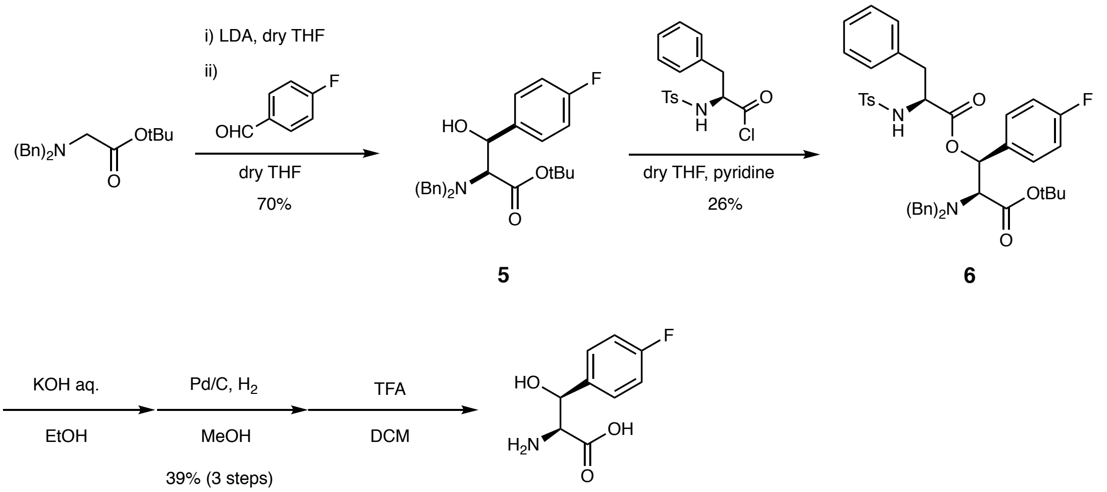

Probe **2**
(L-*erythro* form)

Supplementary Figure 2 | Synthesis of the SHMT NMR probe 2.

The detailed procedures are described in Supplementary Methods section.

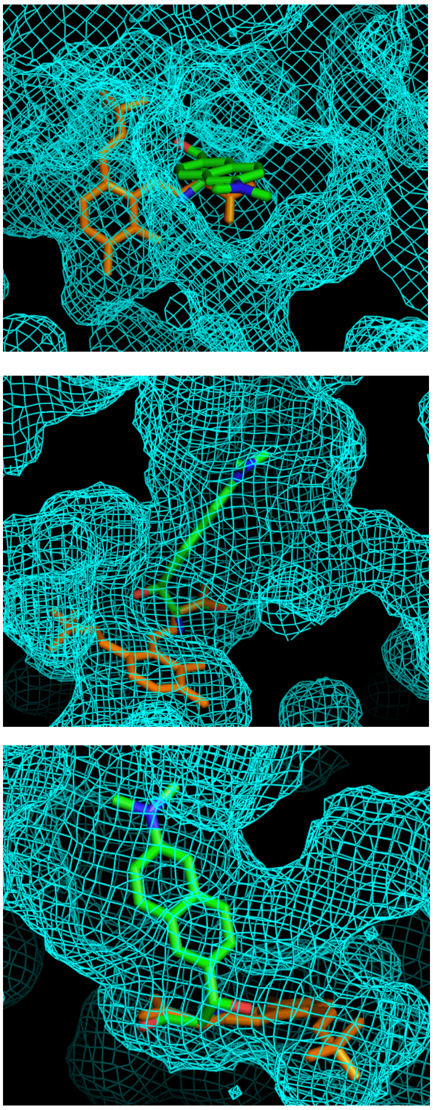

Supplementary Figure 3 | Illustration of probe 1 manually fitted in active site of mSHMT1 (PDB ID: 1EJI).

First, the calculation of global minimum energy conformation of L-*erythro* probe **1** was performed by Spartan'16 (Wavefunction, Inc.) software.[1] Then, the co-crystal structure of PLP-Gly–5-CHO-THF–mSHMT1 (PDB ID: 1EJI) and probe **1** are displayed using the PyMOL software,[2] and 5-CHO-THF is deleted. Finally, L-*erythro* probe **1** was manually placed to the active site of mSHMT1 with PLP-Gly as amino groups and carboxylic groups of Gly and probe **1** were each superimposed. This supports the hypothesis that L-*erythro* form can be accommodated in the substrate pocket. Color code of stick model: oxygen: red; nitrogen: blue; carbon: green; PLP: orange.

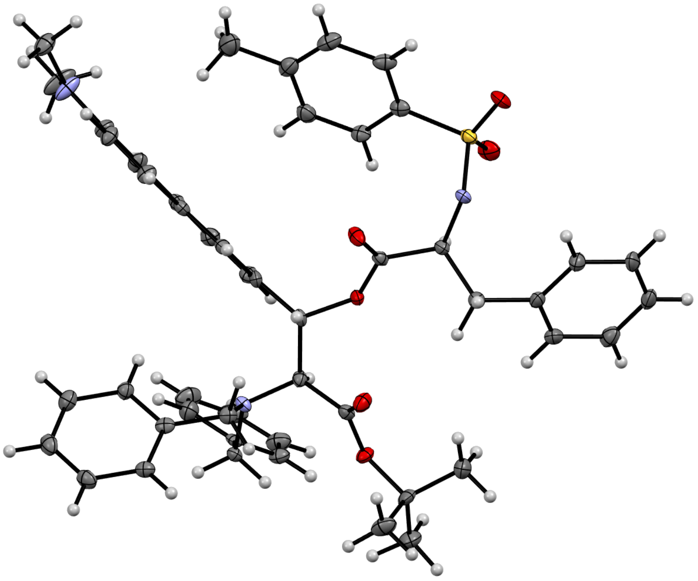

Supplementary Figure 4 | ORTEP drawing of probe 1 L-*erythro* intermediate.

The ORTEP are drawn at 50% probability level. Solvent molecules are omitted for clarity. Color code: oxygen: red; nitrogen: blue; sulfur: yellow; carbon: black; hydrogen: white.

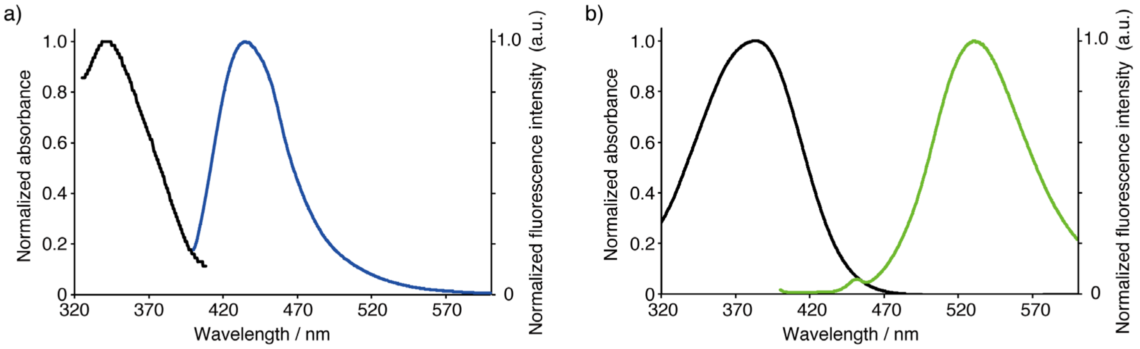

Supplementary Figure 5 | Spectroscopic properties of fluorescent probe 1 and dimethylaminonaphthylaldehyde (DMANA).

Absorbance and fluorescence spectra of (a) probe **1** and (b) DMANA.

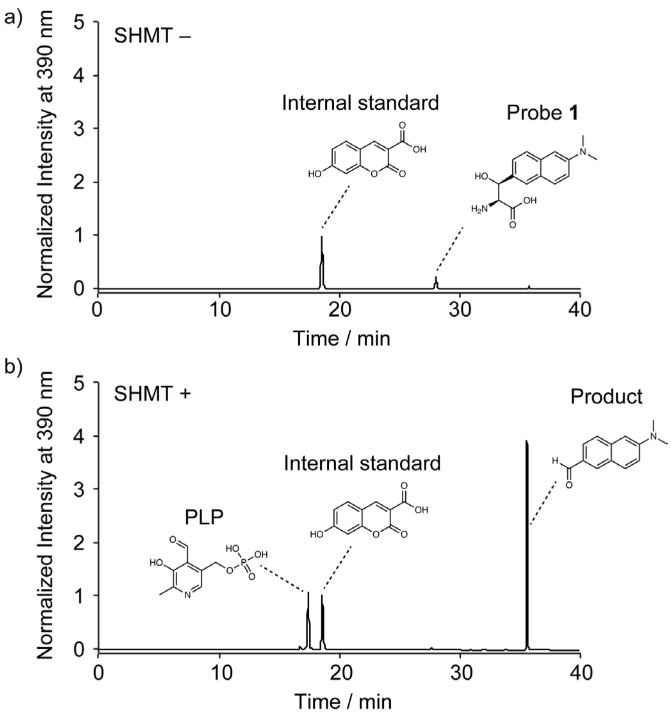

Supplementary Figure 6 | The product confirmation of SHMT-catalyzed probe 1 conversion.

Detection wavelength is 390 nm. 100 μM Probe **1**, 50 mM HEPES buffer (pH 7.5), 100 mM NaCl, 0.5 mM EDTA, 1 mM dithiothreitol (DTT), 1% DMSO, (a) without or (b) with SHMT1.

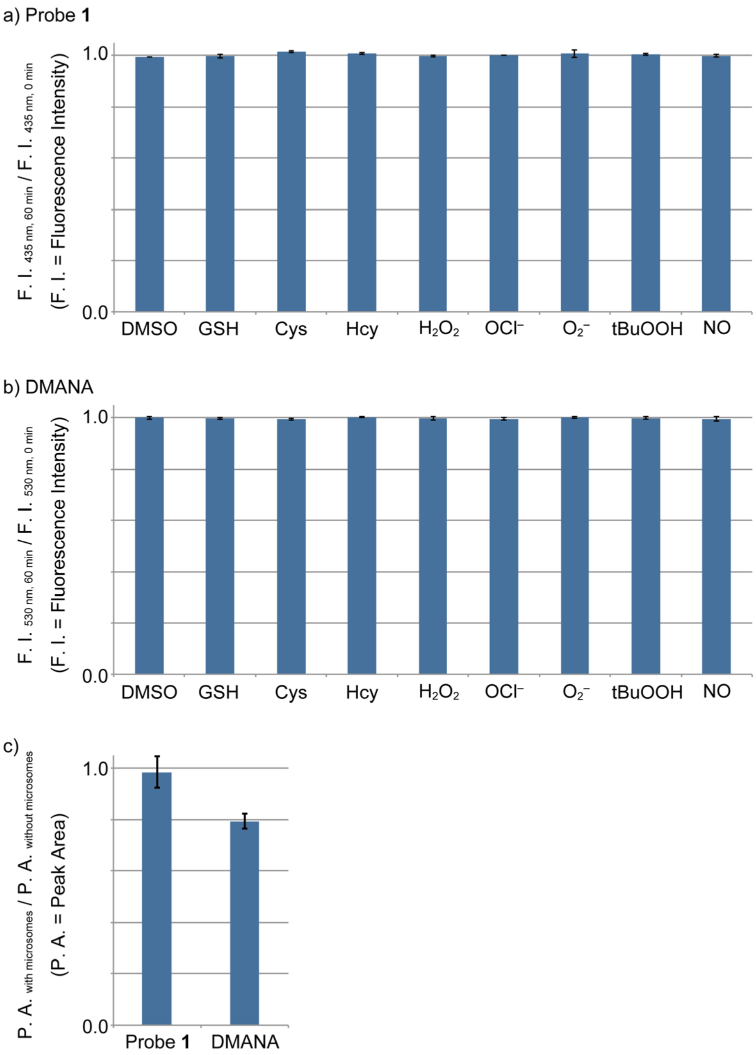

Supplementary Figure 7 | The stability of probe 1 and DMANA.

a) Fluorescence intensity change of probe **1** (1 μM). b) Fluorescence intensity change of DMANA (5 μM). Assay conditions: incubation time for 60 min at 37 ºC. Excitation at 390 nm. 50 mM HEPES buffer (pH 7.5), 100 mM NaCl, 0.5 mM EDTA, 1 mM dithiothreitol (DTT), 1% DMSO. Fluorescence spectra were measured using (a) a SHIMADZU RF-6000 fluorescence spectrometer and using (b) a JASCO FP-6500 fluorescence spectrometer. c) Metabolic stability assays of probe **1** (5 μM) and turned over product DMANA (5 μM). Assay conditions: incubation time for 60 min at 37 ºC. 20 mM HEPES buffer (pH 7.5), rat liver microsomes (SIGMA, M9066-1VL: 200 μg/mL final concentration with 5 μM NADPH). Conversions were monitored using a SHIMADZU HPLC equipped with fluorescence detector RF-10A XL. Probe **1**: excitation at 340 nm, detection at 435 nm. DMANA: excitation at 390 nm, detection at 530 nm. Error bars represent s.d., n = 3. Source data are provided as a Source Data file.

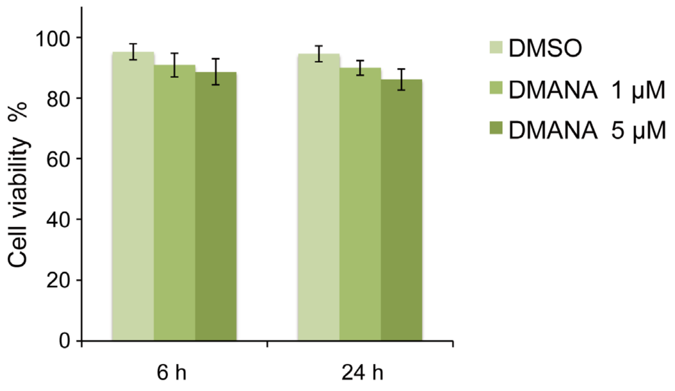

Supplementary Figure 8 | Cytotoxicity evaluation of product DMANA.

HeLa cells were plated in a 6 well plate at a density of 50,000 cells/mL in DMEM media. After incubation for 24 h, each samples were supplemented with either 1 μM or 5 μM DMANA, containing 1% DMSO. Equivalent samples were supplemented with 1% DMSO as a vehicle control. At 6 and 24 hours, cells were dissociated from wells by trypsin, a 10 μL sample was taken from each of the samples and mixed 1:1 with a 0.4% wt/volume trypan blue solution in PBS. Samples were incubated for 1 minute at room temperature before being loaded onto a hemocytometer where live and dead cells were counted. Each sample was made in triplicate for each time point. Error bars represent s.d., $n = 3$. Source data are provided as a Source Data file.

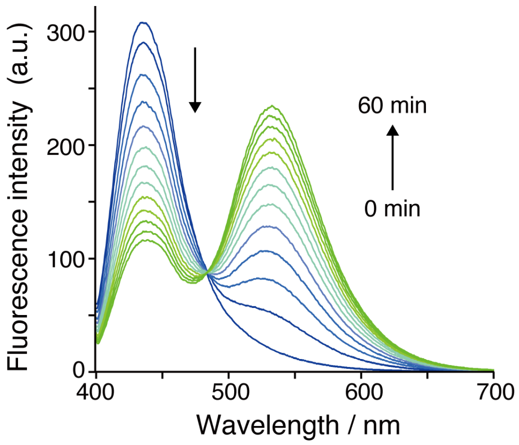

Supplementary Figure 9 | Fluorescence spectral change of probe 1 during the SHMT2-catalyzed reaction.

Excitation at 390 nm. Assay conditions: 5.0 µM probe **1**, 1 unit/mL SHMT2, 50 mM HEPES buffer (pH 7.5), 100 mM NaCl, 0.5 mM EDTA, 1 mM dithiothreitol (DTT), 1% DMSO, 37 ºC. Fluorescence spectra were measured using a JASCO FP-6500 fluorescence spectrometer. Source data are provided as a Source Data file.

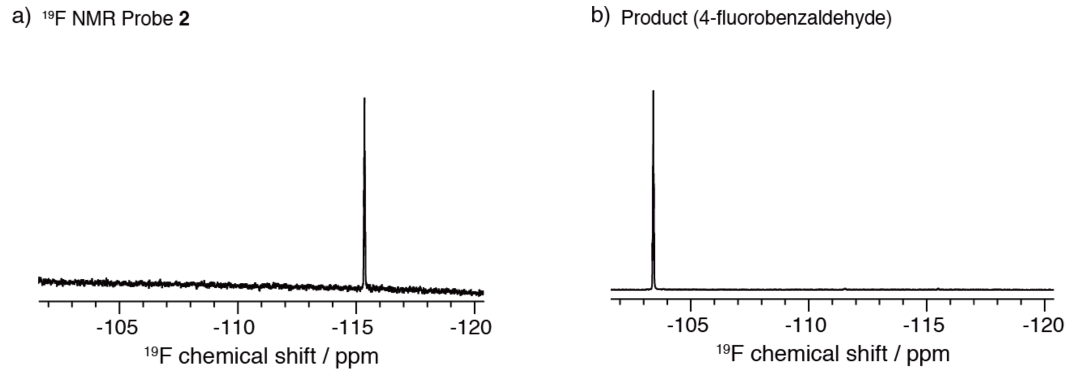

Supplementary Figure 10 | Evaluation of ^{19}F chemical shift.

^{19}F NMR spectra of (a) probe **2** and (b) 4-fluorobenzaldehyde (product).

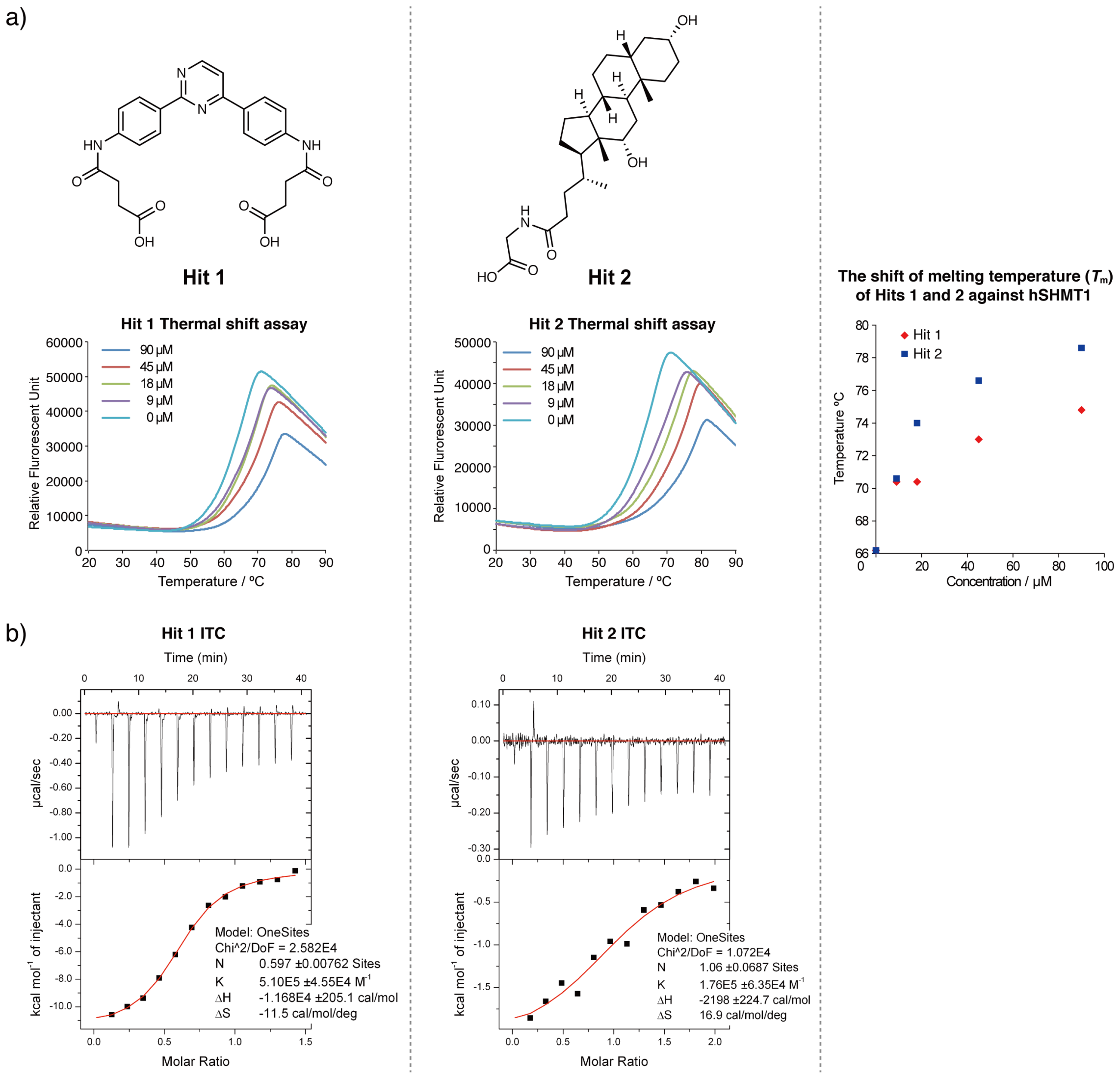

Supplementary Figure 11 | Binding confirmation of Hits 1 and 2 against hSHMT1.

(**a**) Thermal shift assay data and the shift of melting temperature (T_m) of Hits 1 and 2 against hSHMT1. All assays were carried out in 10 mM HEPES (pH 7.5), 100 mM NaCl, 0.5 mM EDTA, 2 mM β-mercaptoethanol, 9 μM (0.5 mg/mL) hSHMT1, 1% DMSO, compound (0, 9, 18, 45, 90 μM), and SYPRO® orange. (**b**) ITC data of Hits 1 and 2 against hSHMT1. All assays were carried out at 37 ºC in 10 mM HEPES,100 mM NaCl, 0.5 mM EDTA, 2 mM β-mercaptoethanol, and 3% DMSO.

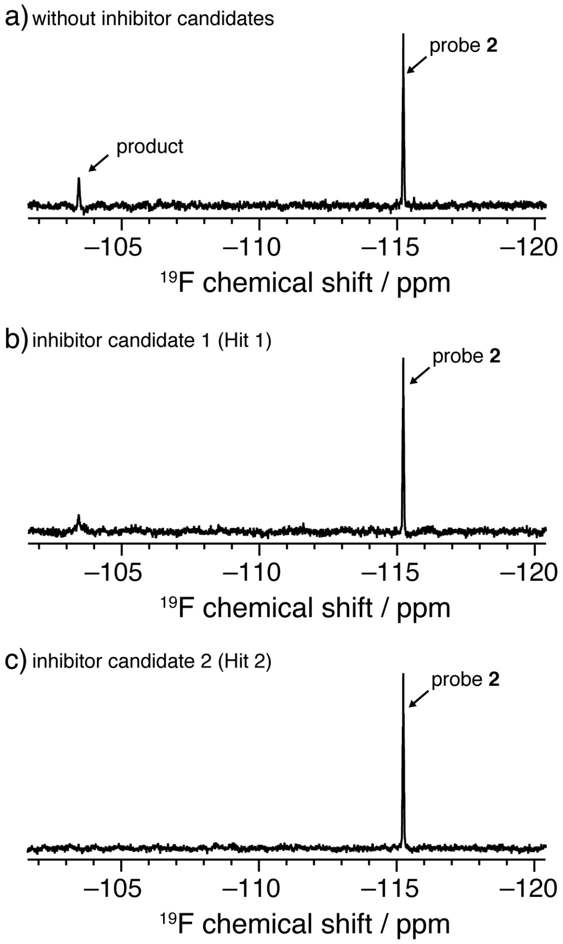

Supplementary Figure 12 | Confirmation of SHMT activity inhibition in mouse liver homogenate by using ^{19}F NMR probe 2.

50 mM HEPES pH 7.5, 100 mM NaCl, 0.5 mM EDTA, 1 mM DTT, 30% D_2O, 16.7 mg proteins/mL, 1 mM ^{19}F NMR probe **2**, 1% DMSO, (a) without or (b, c) with 10 μM inhibitor candidate.

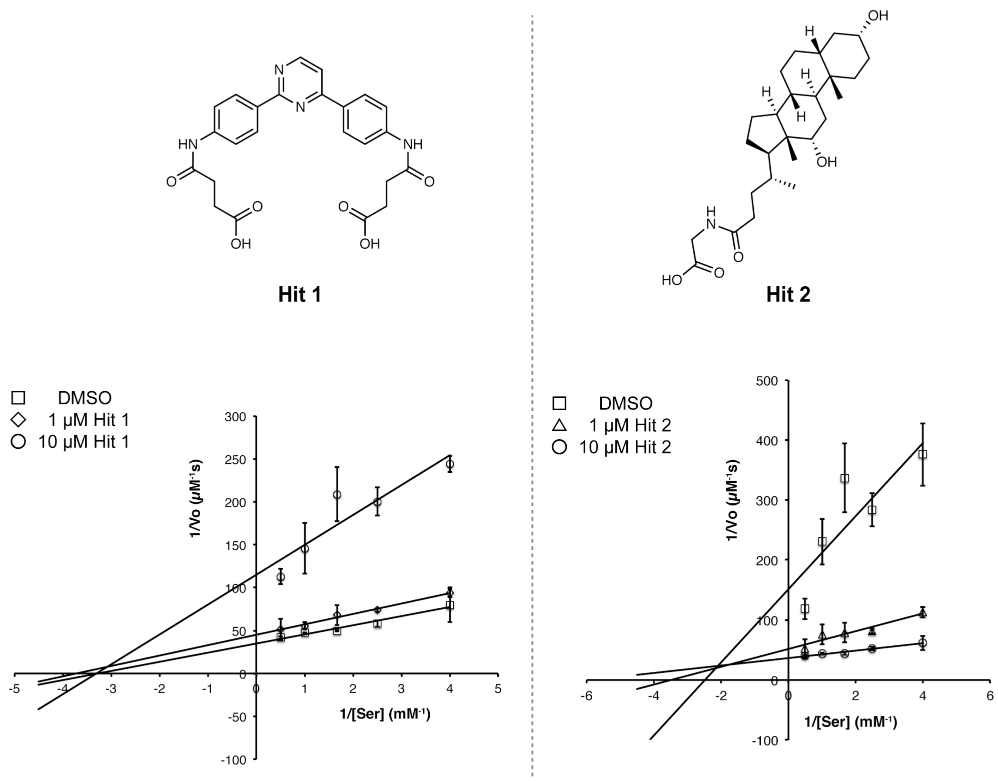

Supplementary Figure 13 | Estimation of inhibition mechanism against hSHMT1.

Lineweaver–Burk plot of hSHMT1 inhibition with altered concentrations of serine. For Hit 1, inhibitor concentrations were 0, 1, and 10 µM. For Hit 2, inhibitor concentrations were 0, 1, and 10 µM. All assays were carried out at 25 ºC in 50 mM HEPES buffer (pH 7.5) containing 0.2 mM DTT and 0.1 mM EDTA. Error bars represent s.d., *n* = 3. Source data are provided as a Source Data file.

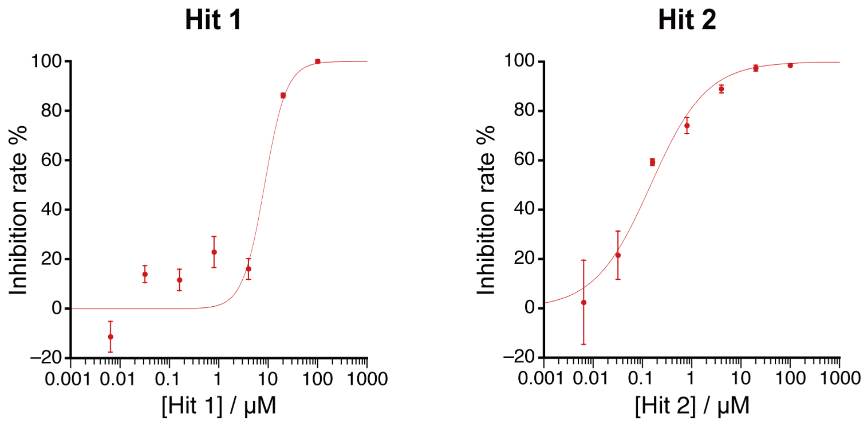

Supplementary Figure 14 | Inhibition properties of Hits 1 and 2 against hSHMT2.

Inhibition rates against hSHMT2 activity were determined by HPLC analysis of Ser–Gly conversion in the presence of various concentrations of Hits 1 and 2. Error bars represent s.d., n = 3. Source data are provided as a Source Data file.

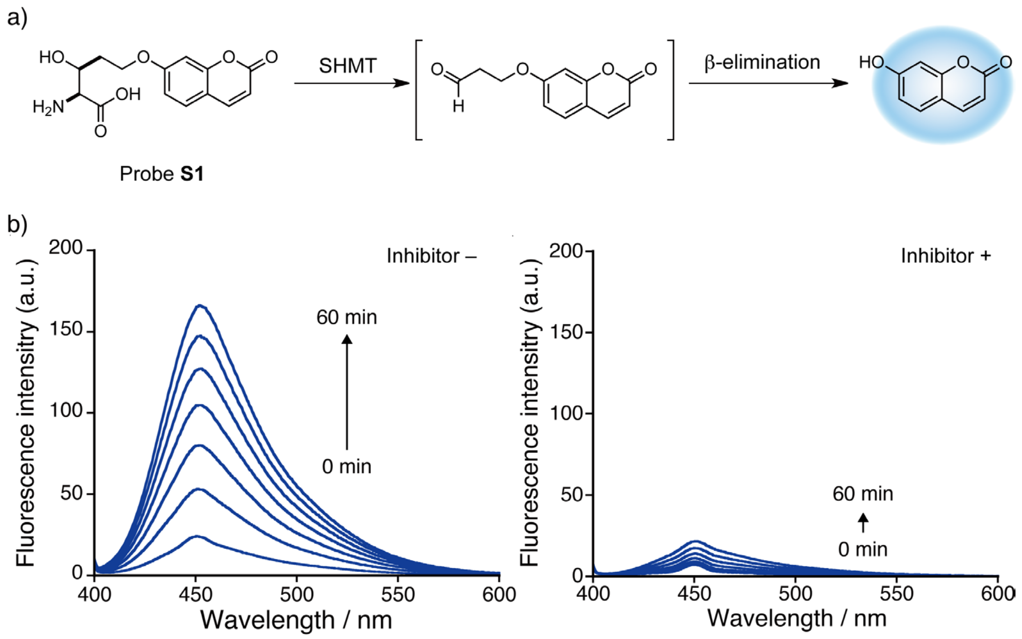

Supplementary Figure 15 | β-elimination-triggered fluorescent probe targeting SHMT.

(a) Schematic illustration of β-elimination-triggered SHMT fluorescent probe **S1**. (b) Fluorescence spectral change of probe **S1** (1 μM) during the SHMT1-catalyzed reaction from 0 to 60 min. Excitation at 390 nm. Assay conditions: 1 unit/mL SHMT1, 50 mM HEPES buffer (pH 7.5), 100 mM NaCl, 0.5 mM EDTA, 1 mM dithiothreitol (DTT), with or without inhibitor (±)-SHIN1 10 μM, 0.2% DMSO, 37 ºC. Fluorescence spectra were measured at 37 ºC using a JASCO FP-6500 fluorescence spectrometer. The reaction buffer (1 unit/mL hSHMT1, 50 mM HEPES pH 7.5, 100 mM NaCl, 0.5 mM EDTA, 1 mM DTT, 0.2% DMSO) was preincubated at 37 ºC for 1 h. The fluorescent probe **S1** was added to the solution and incubated at 37 ºC. Fluorescence emission upon excitation at 390 nm was monitored for 60 min. Source data are provided as a Source Data file.

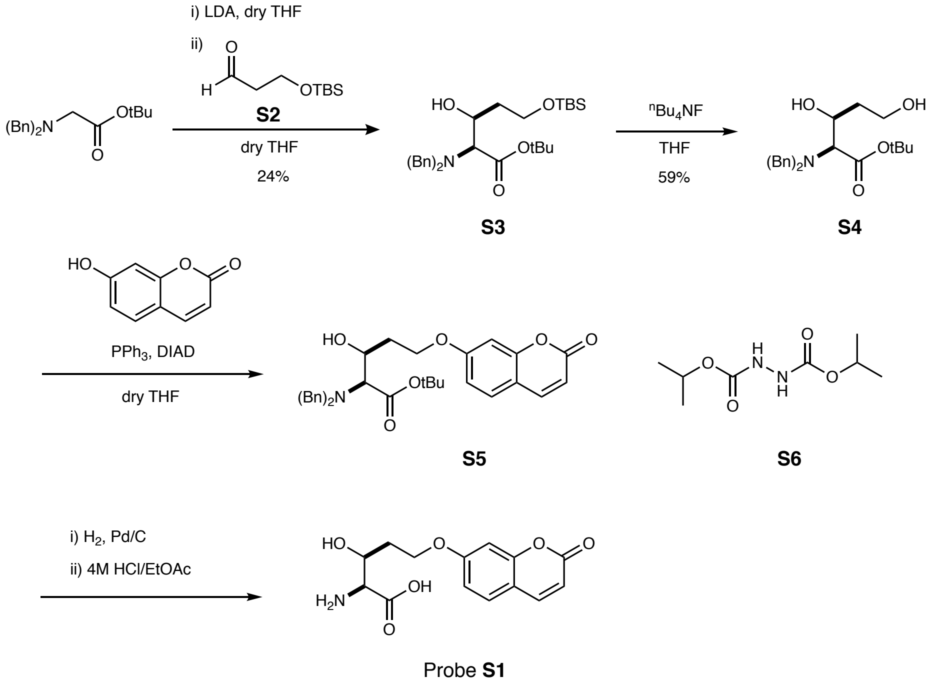

Supplementary Figure 16 | Synthesis of the SHMT fluorescent probe S1.

Supplementary tables

Supplementary Table 1. Data collection and refinement statistics for probe 1 L-*erythro* intermediate.

	probe **1** L-*erythro* intermediate
Crystal System	Triclinic
Space Group	P–1
a (Å)	13.171(3)
c (Å)	14.839(4)
V (Å^3)	2363.6(10)
Z	2
Diffractometer	CCD
μ(mm^{-1})	0.117
Radiation type	Mo $K\alpha$
Radiation wavelength (Å)	0.71075
F(000)	864
Goodness of fit	1.085
Temperature (K)	93
Reflections collected	30920
Independent reflections	8314
R_1 ($I > 2.00\sigma(I)$)[a]	0.0585
wR_2 ($I > 2.00\sigma(I)$)[b]	0.1469

[a] $R_1 = \sum||F_o|-|F_c||/\sum|F_o|$, [b] $wR_2 = [\sum w|F_o^2-F_c^2|^2/\sum w(F_o^2)^2]^{1/2}$

Supplementary Table 2. Optimization of the assay conditions for high-throughput screening.

Details are dscribed in Supplementary Note 1.

Item	*Optimized condition*	*Comments*
Probe concentration	2.5 μM	0.313, 0.625, 1.25, 2.5, and 5 μM were tested.
SHMT1 concentration	0.1 unit/mL	0.025, 0.05, 0.1, and 0.2 unit/mL of hSHMT1 were tested.

Supplementary Methods

Synthesis of fluorescent and ^{19}F NMR probes

Reagents and solvents were purchased from standard suppliers and used without further purification. NMR spectra were measured using a JEOL ECS400 spectrometer or a Bruker Ascend 500 NMR spectrometer. Chloroform-d_1 (7.26 ppm) and methanol-d_4 (3.31 ppm) were used as internal standards for ^{1}H NMR. Chloroform-d_1 (77.0 ppm), methanol-d_4 (49.0 ppm), or dioxane in D_2O (67.2 ppm) was used as the internal standard for ^{13}C NMR. CF_3COOH (–76.5 ppm) was used as the internal standard for ^{19}F NMR. Mass spectra (MS) were measured using a Bruker micrOTOF II (ESI).

Synthesis of 3

To a solution of diisopropylamine (460 μL, 3.3 mmol) in dry THF (4 mL) at −10°C was added 1.6 M solution of *n*-BuLi in hexane (1900 μL, 3.0 mmol) and then the mixture was stirred for 20 min on ice under N_2 atmosphere. *N,N*-dibenzylglycine *tert*-butyl ester (623 mg, 2.0 mmol) in dry THF (4 mL) was added into the solution via cannula at −60°C. After stirring for 10 min, 6-dimethylaminonaphthylaldehyde (438 mg, 2.2 mmol) was added into the solution and stirred for 30 min at −60°C. After quenching with saturated aqueous NH_4Cl, the mixture was extracted with diethyl ether, and then the organic layer was dried with anhydrous Na_2SO_4. After removing the solvent *in vacuo*, the residue was purified by flash column chromatography on silica gel with dichloromethane to give **3**. (699 mg, 68 %). ^{1}H NMR (CD_3OD, 400 MHz) δ = 1.64 (s, 9 H), 3.07 (s, 6 H), 3.38 (d, J = 14.0 Hz, 2 H), 3.52 (d, J = 10.0 Hz, 1 H), 3.83 (d, J = 14.0 Hz, 2 H), 5.09 (d, J = 10.0 Hz, 1 H), 6.86–7.62 (m, 16 H); ^{13}C NMR (CD_3OD, 100 MHz) δ = 28.90, 41.33, 55.93, 68.03, 74.10, 82.64, 107.94, 117.85, 127.20, 127.26, 127.96, 128.04, 128.08, 128.96, 129.83, 130.08, 136.36, 136.67, 140.21, 150.36, 172.25; HRMS (ESI): m/z calc. for $C_{33}H_{39}N_2O_3^+$ $[M+H]^+$ = 511.2955, found = 511.2964.

Synthesis of 4

To a solution of **3** (204 mg, 0.50 mmol) and *N,N*-dimethyl-4-aminopyridine (240 mg, 0.50 mmol) in dry THF (5 mL) on ice was added *N*-(*p*-toluenesulfonyl)-L-phenylalanyl chloride (204 mg, 0.75 mmol) in dry THF (5 mL) via cannula under N_2 atmosphere. After stirring for 14 h at room temperature, the mixture was treated with ice-cold water for 5 min to inactivate carboxylic

chloride. The mixture was extracted with ethyl acetate, and then the separated organic layer was washed with 1N HCl (×2) and brine (×2). After drying over anhydrous Na_2SO_4, the solvent was removed *in vacuo*, and the residue was purified by flash column chromatography on silica gel with hexane-ethyl acetate (5:1) to give **4**. (154 mg, 48 %). ^{1}H NMR (DMSO-d$_6$, 400 MHz) δ = 1.53 (s, 9 H), 2.03 (s, 3 H), 2.53 (m, 1 H), 2.80 (m, 1 H) , 3.08 (s, 6 H), 3.39 (d, J = 14.0 Hz, 2 H), 3.63 (d, J = 10.4 Hz, 1 H), 3.77 (d, J = 14.0 Hz, 2 H), 3.93–3.99 (m, 1 H), 6.08 (d, J = 10.8 Hz, 1 H), 6.69 (d, J = 8.4 H, 2 H), 6.86–7.68 (m, 23 H), 8.18 (d, J = 9.5 Hz, 1 H); ^{13}C NMR (DMSO-d$_6$, 100 MHz) δ = 20.65, 28.10, 37.44, 40.33, 54.09, 57.06, 64.10, 74.42, 81.77, 105.60, 116.48, 125.49, 125.579, 125.722, 125.89, 126.43, 127.07, 127.56, 128.03, 128.09, 128.46, 128.73, 128.96, 129.00, 129.80, 134.82, 136.25, 138.12, 141.87, 148.78, 168.48, 169.22; HRMS (ESI): *m*/*z* calc. for $C_{49}H_{54}N_3O_6S^+$ $[M+H]^+$ = 812.3728, found = 812.3740.

Synthesis of 1

To the solution of **4** (204 mg, 0.25 mmol) in ethanol (3 mL) and THF (3 mL) on ice was added dropwise 5M aqueous NaOH (3 mL). After vigorously stirring for 5 min at room temperature, the organic solvent was removed and then the residue was extracted with chloroform. After drying over anhydrous Na_2SO_4, the organic layer was removed *in vacuo*, and then the residue was used directly in the next step without further purification. (132 mg, quant.) ^{1}H NMR ($CDCl_3$, 400 MHz) δ = 1.67 (s, 9 H), 3.10 (s, 6 H), 3.51 (d, J = 13.0 Hz, 2 H), 3.61 (d, J = 9.2 Hz, 1 H), 3.94 (d, J = 13.0 Hz, 2 H), 5.15 (d, J = 9.2 Hz, 1 H), 6.97–7.62 (m, 16 H). To the solution of hydrolysis product (159 mg, 0.30 mmol) and acetic acid (60 µL, 1.0 mmol) in MeOH (5 mL) was added palladium on carbon (16 mg). The mixture was stirred at room temperature for 28 h under H_2 atmosphere. After removal of the palladium catalyst by filtration through a pad of celite, the filtrate was evaporated. The residue was dissolved with chloroform, and then the organic layer was washed with saturated aqueous $NaHCO_3$ (×3). After drying over anhydrous Na_2SO_4, the organic layer was evaporated and then the residue was used directly in the next step without further purification. 4M HCl/ethyl acetate (3 mL) was added dropwise to the crude residue on ice, and then the solution was stirred at room temperature. After removing the solvent, the crude residue was washed with ethyl acetate and then the obtained solid was dried *in vacuo* to give **1** as the HCl salt (63 mg, 59 %). Before using for experiments, the required amount was purified by preparative HPLC using 0.1% TFA in H_2O/acetonitrile solvent system monitored by absorbance at 220 nm. ^{1}H NMR (CD_3OD, 400 MHz) δ = 3.21 (s, 6 H), 4.36 (d, J = 4.0 Hz, 1 H), 5.46 (d, J = 3.6 Hz, 1 H), 7.48–7.93 (m, 6 H); ^{13}C NMR (CD_3OD, 100 MHz) δ =

43.82, 60.17, 72.11, 113.23, 118.53, 126.22, 126.69, 128.59, 130.86, 131.07, 135.50, 135.80, 146.46, 169.20; HRMS (ESI): *m*/*z* calc. for $C_{15}H_{19}N_2O_3^+$ $[M+H]^+$ = 275.1390, found = 275.1404.

Synthesis of 5

To a solution of diisopropylamine (930 μL, 6.9 mmol) in dry THF (8 mL) at −78°C was added 1.6 M solution of *n*-BuLi in hexane (3750 μL, 6.0 mmol) and then the mixture was stirred for 20 min at −78°C under N_2 atmosphere. After *N,N*-dibenzylglycine *tert*-butyl ester (935 mg, 3.0 mmol) in dry THF (6 mL) was added into the solution via cannula and stirred for 10 min, *p*-fluorobenzaldehyde (465 μL, 4.4 mmol) was added into the solution and stirred for 30 min kept at −78°C. After quenching with saturated aqueous NH_4Cl, the mixture was extracted with ethyl acetate, and then the organic layer was dried with anhydrous Na_2SO_4. The solvent was removed *in vacuo*, and the residue was purified by flash column chromatography on silica gel with hexane-ethyl acetate (9:1) to give **5** as a white solid (909 mg, 70 %). ^{1}H NMR ($CDCl_3$, 400 MHz) δ = 1.63 (s, 9 H), 2.81 (d, J = 3.6 Hz, 1 H), 3.44 (d, J = 9.2 Hz, 1 H), 3.47 (d, J = 14.0 Hz, 2 H), 3.86 (d, J = 13.6 Hz, 2 H), 4.97 (m, 1 H), 6.92–7.23 (m, 14 H); ^{13}C NMR ($CDCl_3$, 100 MHz) δ = 28.58, 55.00, 66.41, 72.10, 82.39, 114.79 (d, $^2J_{CF}$ = 20.9 Hz), 127.04, 128.12, 128.80, 129.35 (d, $^3J_{CF}$ = 8.6 Hz), 136.72 (d, $^4J_{CF}$ = 1.9 Hz), 138.35, 162.44 (d, $^1J_{CF}$ = 244 Hz), 171.67; ^{19}F NMR ($CDCl_3$, 376 MHz) δ = −114.57 (tt, $^3J_{HF}$ = 8.6 Hz, $^4J_{HF}$ = 5.6 Hz); HRMS (ESI): *m*/*z* calc. for $C_{27}H_{30}FNNaO_3^+$ $[M+Na]^+$ = 458.2102, found = 458.2102.

Synthesis of 6

To a vigorously stirred solution of **5** (175 mg, 0.40 mmol) in dry THF (4.6 mL) was added pyridine (1.0 mL, 12.5 mmol) and then *N*-(*p*-toluenesulfonyl)-L-phenylalanyl chloride (562 mg, 1.7 mmol) in dry THF (13 mL) via cannula at ambient temperature under N_2 atmosphere. The solution was stirred for 19 h at 60℃. The mixture was treated with ice-cold water (3 mL) to inactivate carboxylic chloride. The whole was partitioned between ethyl acetate and brine. The separated organic layer was washed with 0.1 M aqueous HCl, brine, and saturated aqueous $NaHCO_3$ and dried with anhydrous Na_2SO_4. The solvent was removed *in vacuo*, and the residue was purified by flash column chromatography on silica gel with hexane-ethyl acetate (7:1) to give **6** as a white solid. (75.8 mg, 26 %). ^{1}H NMR ($CDCl_3$, 400 MHz) δ = 1.61 (s, 9 H), 2.26 (s, 3 H), 2.84 (dd, J = 6.0, 13.6 Hz, 1 H), 2.99 (dd, J = 5.6, 13.6 Hz, 1 H), 3.38 (d, J = 13.4 Hz, 2 H), 3.65 (d, J = 11.2 Hz, 1 H), 3.82 (d, J = 13.2 Hz, 2 H), 4.11 (m, 1 H), 4.77 (d, J = 9.6 Hz, 1

H), 5.92 (d, J = 10.8 Hz, 1 H), 6.80–7.32 (m, 23 H); ^{13}C NMR ($CDCl_3$, 100 MHz) δ = 21.36, 28.70, 38.82, 54.66, 55.92, 63.54, 74.50, 82.57, 114.81 (d, $^2J_{CF}$ = 22.0 Hz), 126.52, 127.12, 127.25, 128.18, 128.44, 128.93, 129.20, 129.83, 130.31 (d, $^3J_{CF}$ = 8.6 Hz), 132.55 (d, $^4J_{CF}$ = 2.9 Hz), 134.42, 136.55, 137.87, 143.07, 162.61 ($^1J_{CF}$ = 245 Hz), 168.89, 169.36; ^{19}F NMR ($CDCl_3$, 376 MHz) δ = −112.90 (tt, $^3J_{HF}$ = 8.1 Hz, $^4J_{HF}$ = 5.6 Hz); HRMS (ESI): *m*/*z* calc. for $C_{43}H_{46}FN_2O_6S^+$ $[M+H]^+$ = 737.3055, found = 737.3030.

Synthesis of 2

To a solution of **6** (75.8 mg, 0.10 mmol) in EtOH (6 mL) was added ca. 5M aqueous KOH (6 mL), and the whole was heated at 40℃ for 2 h. The solution was partitioned between ethyl acetate and brine, and the organic layer was separated. The organic layer was dried with anhydrous Na_2SO_4 and the solvent was removed *in vacuo*. To deprotect benzyl group, the crude residue was dissolved in MeOH (10 mL), and palladium on carbon (15 mg) was added to the solution. The solution was stirred at ambient temperature for 16 h. After removal of the palladium catalyst by filtration through a pad of celite, the filtrate was evaporated. To deprotect *tert*-butyl group, the crude residue was dissolved in TFA (2 mL) and DCM (2 mL) at 0℃, and then the solution was stirred at ambient temperature for 17 h. After removing the solvent, the crude was purified by preparative HPLC using 0.1% TFA in H_2O/acetonitrile solvent system monitored by absorbance at 220 nm. The fraction was freeze-dried to give the white solid **2** (8.3 mg, 41 %) as the trifluoroacetic acid salt. The yield was determined by ^{1}H NMR using 1,4-dioxane as an internal standard. ^{1}H NMR (D_2O, 400 MHz) δ = 3.98 (d, J = 4.0 Hz, 1 H), 5.24 (d, J = 4.4 Hz, 1 H), 7.02–7.07 (m, 2 H), 7.25–7.30 (m, 2 H); ^{13}C NMR (D_2O, 100 MHz) δ = 59.52, 70.44, 115.62 (d, $^2J_{CF}$ = 21.9 Hz), 128.18 (d, $^3J_{CF}$ = 8.5 Hz), 132.85 (d, $^4J_{CF}$ = 2.9 Hz), 162.73 (d, $^1J_{CF}$ = 243 Hz), 170.08; ^{19}F NMR (D_2O, 376 MHz) δ = −113.98 (tt, $^3J_{HF}$ = 9.0 Hz, $^4J_{HF}$ = 5.6 Hz); HRMS (ESI): *m*/*z* calc. for $C_9H_{11}FNO_3^+$ $[M+H]^+$ = 200.0717, found = 200.0726.

Synthesis of S3

To a solution of diisopropylamine (80 μL, 0.56 mmol) in dry THF (2 mL) at −78 °C was added 1.6 M solution of *n*-BuLi in hexane (320 μL, 0.51 mmol) and then the mixture was stirred with gradually warming to room temperature over 30 min under N_2 atmosphere. *N,N*-dibenzylglycine *tert*-butyl ester (106 mg, 0.34 mmol) in dry THF (2 mL) was added into the solution via cannula at 0 °C. After stirring for 10 min, 3-((*tert*-butyldimethylsilyl)oxy)propanal[3] (70.2 mg, 0.37 mmol) (**S2**) in dry THF (2 mL) was added into the solution, and then the reaction mixture was

allowed to warm to room temperature with stirring for 12 h. After quenching with saturated aqueous NH_4Cl, the mixture was extracted with ethyl acetate. The organic layer was washed with brine and dried with anhydrous Na_2SO_4. After filtration and removing the solvent under reduced pressure, the residue was purified by flash column chromatography on silica gel with hexane-ethyl acetate (49:1 to 45:5) to give **S3** (41.5 mg, 24 %). ^{1}H NMR ($CDCl_3$, 400 MHz) δ = 0.01 (s, 3 H), 0.03 (s, 3H), 0.85 (s, 9H), 1.33–1.41 (m, 1H), 1.61 (s, 9H), 2.11–2.19 (m ,1H), 3.13 (d, J = 9.6 Hz, 1H), 3.28 (d, J = 3.6 Hz, 1H), 3.48 (d, J = 13.6, 2H), 3.67–3.76 (m, 2H), 3.94 (d, J = 13.6 Hz, 2H), 4.19–4.24 (m, 1H), 7.22–7.36 (m, 10 H); ^{13}C NMR ($CDCl_3$, 100 MHz) δ = –5.37, –5.32, 18.33, 26.06, 28.79, 35.21, 55.55, 61.59, 65.93, 69.21, 81.75, 127.28, 128.41, 129.32, 139.33, 171.33; HRMS (ESI): m/z calc. for $C_{29}H_{46}NO_4Si^+$ $[M+H]^+$ = 500.3191, found = 500.3189.

Synthesis of S4.

To **S3** (438 mg, 0.878 mmol) in a 6 mL vial equipped with a magnetic stirring bar was added 1.0 M solution of tetrabutylammonium fluoride of THF (1.31 mL, 1.31 mmol). The reaction mixture was stirred at room temperature for 2 h. After addition of water, the resulting solution was extracted with ethyl acetate. The organic layer was combined, washed with brine, dried over anhydrous Na_2SO_4, filtrated, and evaporated. The residue was purified by flash column chromatography on silica gel with hexane-ethyl acetate (13:7 to 8:12) to give **S4** (198 mg, 59%). ^{1}H NMR ($CDCl_3$, 400 MHz) δ = 1.37–1.46 (m, 1H), 1.62 (s, 9H), 2.06–2.13 (m, 1H), 2.51 (brs, 1H), 3.08 (brs, 1H), 3.15 (d, J = 9.6 Hz, 1H), 3.53 (d, J = 13.2 Hz, 2H), 3.63–3.65 (m, 2H), 3.89 (d, J = 13.6 Hz), 4.13–4.19 (m, 1H), 7.23–7.35 (m, 10H); ^{13}C NMR ($CDCl_3$, 100 MHz) δ = 28.79, 34.64, 55.78, 61.47, 65.71, 70.39, 82.53, 127.50, 128.53, 129.33, 138.90, 172.04; HRMS (ESI): m/z calc. for $C_{23}H_{32}NO_4^+$ $[M+H]^+$ = 386.2326, found = 386.2324.

Synthesis of S5.

To a 10 mL Schlenk tube equipped with a magnetic stirrer bar were added **S4** (96.5 mg, 0.25 mmol), 7-hydroxycumarin (44.9 mg, 0.28 mmol), triphenylphosphine (72.1 mg, 0.27 mmol). The vessel was filled with nitrogen gas, and then added dry THF (1.3 mL). After cooling in an ice bath followed by addition of diisopropyl azodicarboxylate (DIAD, 53.5 μL, 0.28 mmol), the reaction mixture was stirred and gradually allowed to room temperature over 5 h. After addition of ethyl acetate and water for quenching, the result mixture was extracted with ethyl acetate, and then organic phases were combined, washed with saturated $NaHCO_3$ aqueous solution and brine,

dried over Na_2SO_4, filtrated, and evaporated. The residue was purified by flash column chromatography on silica gel with hexane-ethyl acetate (4:1 to 3:2) to give a mixture of **S5** and a by-product **S6** generated from DIAD (72.8 mg, **S5**/**S6** = 1:9.5). The mixture was used for the next step without further purification. ^{1}H NMR ($CDCl_3$, 400 MHz) δ = 1.46–1.53 (m, 1H), 1.63 (s, 9H), 2.41–2.48 (m, 1H), 2.71 (brs, 1H), 3.14 (d, J = 9.2 Hz, 1H), 3.52 (d, J = 13.2 Hz, 2H), 3.89–3.95 (m, 1H), 3.91 (d, J = 13.2 Hz, 2H), 4.07–4.13 (m, 1H), 4.19–4.23 (m, 1H), 6.25 (d, J = 9.6 Hz, 1H), 6.75–6.79 (m, 2H), 7.22–7.35 (m, 11H), 7.62 (d, J = 9.6 Hz, 1H); ^{13}C NMR ($CDCl_3$, 100 MHz) δ = 28.83, 29.88, 32.44, 55.73, 65.35, 65.56, 66.94, 82.62, 101.69, 112.64, 113.17, 127.54, 128.54, 128.83, 129.47, 138.90, 143.62, 156.07, 161.57, 162.41, 171.99; HRMS (ESI): *m*/*z* calc. for $C_{32}H_{36}NO_6^+$ $[M+H]^+$ = 530.2537, found = 530.2539.

Synthesis of S1.

To a 10 mL Schlenk tube equipped with a magnetic stirrer bar were added the obtained mixture of **S5** and **S6** (36.4 mg), and palladium on carbon (10%wt, 2.0 mg). The vessel was filled with nitrogen gas and then added dry methanol (1.0 mL). Then, the vessel was evacuated, filled back hydrogen gas from a balloon, and stirred at room temperature. After 22 h, the reaction solution was filtrated through Celite® and evaporated. The residue was subjected to hydrogen chloride (4N solution of ethyl acetate, 1.0 mL) for 5.5 h, and then evaporated. The resulting crude was purified by preparative HPLC using 0.1% TFA in H_2O/acetonitrile solvent system monitored by absorbance at 320 nm. The desired product **S1** was obtained as a white power of TFA salt (0.5 mg, 1% yield from **S4**). ^{1}H NMR (D_2O, 400 MHz) δ = 2.08–2.12 (m, 2H), 3.99 (s, 1H), 4.26–4.36 (m, 2H), 4.41–4.45 (m, 1H), 6.37 (d, J = 9.6 Hz, 1H), 7.04–7.06 (m, 2H), 7.63 (d, J = 9.6 Hz, 1H), 8.01 (d, J = 9.6 Hz, 1H); ^{13}C NMR (D_2O, 100 MHz) δ = 30.86, 31.68, 59.32, 65.65, 102.13, 112.40, 113.80, 114.12, 130.24, 146.72, 155.77, 162.39, 165.58, 171.35; HRMS (ESI): *m*/*z* calc. for $C_{14}H_{16}NO_6^+$ $[M+H]^+$ = 294.0972, found = 294.0969.

Preparation of plasmid for recombinant hSHMT1

Plasmid for recombinant hSHMT1 was prepared using pET28a(+) plasmid with following primers.

SHMT1 forward: 5'-CAC CCA TAT GAC GAT GCC AGT CAA CGG G-3' (Xho1)

SHMT1 reverse: 5'-CAC CCT CGA GTT AGA AGT CAG GCA GGC CAG G-3' (Nde1)

Preparation of plasmid for recombinant hSHMT2

Plasmid for recombinant hSHMT2 was prepared using hSHMT2(A269T) plasmid with following primers.

SHMT2 mutation forward: 5'-CAC AAG ACT CTT CGA GGG GCC AGG-3'

SHMT2 mutation reverse: 5'-ATG AGC CCT GAC CTG GCC CCT-3'

1st screening (large-scale high-throughput screening)

We conducted high-throughput screening of chemical library (208,233 compounds) supplied by Drug Discovery Initiative, The University of Tokyo. Stock solutions of compounds was diluted with 2 μL of HEPES buffer (50 mM, pH 7.5) containing 100 mM NaCl, 0.5 mM EDTA, 1 mM DTT, 0.005% Tween20, 5.0 μM probe **1**, using a Multi-Drop device (Thermo Scientific). Then, 2 μL of hSHMT1 (0.2 units/mL) in the HEPES buffer was added to the each well, and the plates were centrifuged (240 g, 3 min). Fluorescence intensity of each well was measured (λ_{ex} = 390 nm, λ_{em} = 530 nm) using microplate reader (PHERAster, BMG Labtech). The plates were incubated at 25 ºC for 2 hours. Inhibition rate % = (1-(Measured value - Minimum response)/(Maximum response - Minimum response)) × 100, where maximum response was calculated from the results of wells without the inhibitors (n = 16), and minimum response was calculated from the results of wells without hSHMT1 and the inhibitors (n = 32). 515 compounds that inhibition rate is more than 50% were picked up.

1st screening (confirmation assay)

The same screenings were performed (N = 4) against 515 hits to confirm reproducibility. 224 compounds that have good reproducibility and inhibition rate of more than 30 % were picked up.

1st screening (dose-response test)

The 224 compounds, which passed the confirmation assay, were subjected to further HTS to evaluate their inhibitory activity at five different concentrations. 21 compounds showed dose-dependent inhibition, and their IC_{50} value was estimated as lower than 5 μM.

2nd screening (inhibition assay of Ser-Gly conversion)

The 21 compounds were subjected to HPLC-based assay to confirm that they inhibit Ser-Gly conversion. 0.5 μL of the hit compounds (1 mM in DMSO) were applied to each well of 96 well plate. 5 μL of substrate solution (10 mM HEPES pH 7.5, 100 mM NaCl, 0.5 mM EDTA, 1 mM DTT, 1 mM Ser) was added. Then 25 μL of enzyme solution (10 mM HEPES pH 7.5, 100 mM NaCl, 0.5 mM EDTA, 1 mM DTT, 0.1 U/mL hSHMT1) was added to start the enzymatic reaction. The plate was incubated at 37 ºC for 30 minutes. 40 μL of 50 mM HCl was added to stop the reaction. 50 μL of 200 mM borate buffer was added. 52.5 μL of the solution was sampled for NBD-F labeling. 22.5 μL of 20 mM NBD-F was added and incubated at 60 ºC for 5 min. 1 M HCl was added and cooled on the ice. 20 μL of 200 μM 5'-carboxyfluorescein was added as internal standard. 50 μL of the solution was applied to HPLC analysis with detection at Abs_{470}. 8 compounds showed inhibition of Ser-Gly conversion.

3rd screening (counter assay against DHFR)

The 8 compounds were subjected to counter assay against dihydrofolate reductase (DHFR) derived from *E-coli* to confirm selectivity of inhibitory effect. 1 μL of hit compounds (1 mM in DMSO) were added to each well of 96 well plate, 50 μL of enzyme solution (100 mM Tris (pH7.4), 100 mM NaCl, 10 mM DTT, 120 μM NADPH, 34 μunits/mL thioredoxin fused-eDHFR) and 50 μL of substrate solution (100 mM Tris (pH 7.4), 100 mM NaCl, 10 mM DTT, 100 μM dihydrofolate) were added. The plate was incubated at 25 ºC for an hour, and then Abs_{340} was measured by plate reader. 7 compounds that showed less than 20% inhibition were picked up.

1 activity unit of eDHFR was defined as the amount of enzyme consuming 1 μmol of NADPH for 1 hour at 25 ºC. Reaction conditions: 100 mM Tris-HCl (pH 7.4), 100 mM NaCl, 10 mM DTT, 60 μM NADPH, 50 μM dihydrofolate.

3rd screening (counter assay against ALT)

0.5 μL of the hit compounds (2 mM in DMSO) was added to substrate solution (100 mM HEPES (pH 7.5), 150 μM NADH, 0.5 mM L-alanine, 0.5 mM 2-oxoglutarate). After adding 1 μL of alanine transaminase (ALT, purchased from Oriental Yeast, 1.6 μM) and 2 μL of lactate dehydrogenase (LDH, purchased from Oriental Yeast, 12.3 μM), the reaction was monitored at absorbance of 340 nm at 25 ºC. Absorbance was measured 10 min after enzyme addition. Inhibition rate was calculated by 0% and 100% control.

4th screening (DSF, ITC)

The 3 compounds were subjected to DSF and ITC measurements.

DSF analysis was performed on an Auto-iTC200 (GE healthcare). All assays were carried out in 10 mM HEPES (pH 7.5), 100 mM NaCl, 0.5 mM EDTA, 2 mM β-mercaptoethanol, 9 μM (0.5 mg/mL) hSHMT1, 1% DMSO, compound (0, 9, 18, 45, 90 μM), and SYPRO® orange.

ITC analysis was performed on an Auto-iTC200 (GE healthcare). The measurements were conducted at 37 ºC. A solution of hit compound (300 μM) in 10 mM HEPES, 100 mM NaCl, 0.5 mM EDTA, 2 mM β-mercaptoethanol, and 3% DMSO was injected stepwise to a solution of hSHMT1 (41.2 μM in Hit 1, 29.6 μM in Hit 2) dissolved in the same solvent system. The measured heat flow was recorded as function of time and converted into enthalpies (ΔH) by integration of the appropriate reaction peaks. The binding parameters (K_{app}, ΔH, ΔS, n) were evaluated by applying one-site model using the software Origin7 (GE healthcare).

5th screening (^{19}F NMR using mouse liver homogenate)

BALB/c nude mice (male, 13 weeks old) was sacrificed, and the liver was collected and washed with PBS buffer. The washed liver was added in liquid nitrogen immediately after collection, and kept at –80 ºC. To prepare the lysate, freezed tissue (1316 mg) was put in a shaft-generator homogenizer and twice volumes of PBS(–) buffer (pH 7.5) were added. The tissues were homogenized, and the homogenate was put in 1.5 mL plastic tubes and centrifuged (10,000g × 15 min at 4 ºC). The supernatant was collected as the lysate, and the protein concentration was determined with standard BCA assay. The lysates were aliquoted and kept at –80 ºC. ^{19}F NMR probe **2** (final conc. 1 mM) was added to the mouse liver

homogenate (16.7 mg proteins/mL in PBS), incubated at 37 ºC, and then subjected to ^{19}F NMR analysis.

Inhibition assay of Ser-Gly conversion

25 μL of substrate solution containing Ser (2 mM) and THF (0.8 mM) was mixed with 25 μL of enzyme solution (hSHMT1 0.2 μM, or hSHMT2 0.36 μM) with or without hit compounds. The solution was incubated at 25 ºC for 30 min. Then 40 μL of 50 mM HCl was added to stop the reaction, and 50 μL of 200 mM borate buffer (pH 9.5) was added. 52.5 μL of the mixture was sampled to the reaction with 22.5 μL of 20 mM NBD-F in acetonitrile. After incubation at 60 ºC for 5 min, 5 μL of 1 M HCl was added to quench the reaction. 10 μL of the resultant solution was subjected to HPLC analysis. HPLC analyses were performed on a COSMOSIL® 5C18-MS-II (4.6 mm × 150 mm) column (Nacalai tesque) using a HPLC system composed of a pump (LC-20AT, Shimadzu) and a detector (RF-10A, Shimadzu). Elution was done with eluent A (0.1 M acetic acid/triethylamine buffer (pH 7.0)) and eluent B (acetonitrile). Samples were analyzed using HPLC with a linear gradient: 0–2.5 min, 12% B; 2.5–8.0 min, 12-37% B; 8.0–8.5 min, 100% B; 8.5–14.5 min, 100% B; 14.5–15.0 min, 12% B; 15.0–25.0 min, 15% B at a flow rate of 1.0 mL/min. HPLC charts were monitored at fluorescence intensity at 540 nm upon excitation at 490 nm.

Preparation of rat liver homogenate

Sprague-Dawley rat (male, 8 weeks old) was sacrificed, and the liver was collected and washed with PBS buffer. The washed liver was added in liquid nitrogen immediately after collection, and kept at –80 ºC. To prepare the lysate, freezed tissue (762 mg) was put in a shaft-generator homogenizer and twice volumes of PBS(–) buffer (pH 7.5) were added. The tissues were homogenized, and the homogenate was put in 1.5 mL plastic tubes and centrifuged (10,000g × 15 min at 4 ºC). The supernatant was collected as the lysate, and the protein concentration was determined with standard BCA assay. The lysates were aliquoted and kept at –80 ºC.

Measurement of SHMT activity using rat liver homogenate

Rat liver homogenate (250 µg/mL) in the reaction buffer (10 mM HEPES pH 7.5, 100 mM NaCl, 0.5 mM EDTA, 1 mM DTT) was preincubated at 25 ºC for an hour. The solution was added to 0.5 µL of inhibitor (1 mM or 0.2 mM in DMSO). To the solution, 25 µL of substrate solution (10 mM HEPES pH 7.5, 100 mM NaCl, 0.5 mM EDTA, 1 mM DTT, 1 mM Ser, 0.8 mM THF) was added to start the reaction. The reaction was performed at 25 ºC for 1 hour and quenched by adding 40 µL of 50 mM HCl. To the solution, 50 µL of 200 mM borate buffer (pH 9.5) was added. Insolubles were removed by centrifugation (14,000g, 30 min, at 25 ºC) using Amicon ultra filter (3000 MWCO, Merck Millipore). 52.5 µL of the resultant mixture was labeled with NBD-F and analyzed by the same methods as inhibition assay of Ser–Gly conversion.

Supplementary Notes

Supplementary Note 1.

Z' factor was calculated from the fluorescence at 530 nm (λex = 390 nm) measured by a plate reader using a 1536 well plate. The quality assessment indexes for screening system showed good values when the probe concentration was 2.5 μM, the enzyme concentration was 0.1 units/mL, and the incubation time was 2 hours (The reaction buffer: 50 mM HEPES (pH 7.5), 100 mM NaCl, 0.5 mM EDTA, 1 mM DTT, 0.005% Tween20). The obtained indexes in this condition are as follows; CV100% = 2.9%, CV0% = 2.5%, S/B = 6.5, Z' = 0.88.

Supplementary References

(1) Hehre, W., Ohlinger, S. The Spartan'16 Tutorial and User's Guide, Wavefunction, Inc., Irvine, CA, USA (2016).

(2) DeLano, W. L. The Pymol User's Manual. Delano Scientific, San Carlos, CA, USA (2002).

(3) Yamada, T., Miki, S., Ul'Husna, A., Michikawa, A. & Nakatani, K. Synthesis of Naphthyridine Carbamate Dimer (NCD) Derivatives Modified with Alkanethiol and Binding Properties of G–G Mismatch DNA, *Org. Lett.* **19**, 4163–4166 (2017).
